# Editorial: Neuroinflammatory diseases of domestic animals

**DOI:** 10.3389/fvets.2022.1039155

**Published:** 2022-09-29

**Authors:** John H. Rossmeisl, Andrea Tipold

**Affiliations:** ^1^Department of Small Animal Clinical Sciences, Virginia Tech, Blacksburg, VA, United States; ^2^University of Veterinary Medicine Hannover, Hanover, Germany

**Keywords:** meningoencephalitis, meningitis, vasculitis, neuropathology, immunology

Inflammatory diseases account for a significant proportion of the nervous system disorders encountered in veterinary practice, are challenging to diagnose, and remain an important cause of morbidity and death in dogs and cats (1–3). Since in many of these diseases the etiopathogenesis and local and systemic immune reactions are poorly characterized, this Research Topic aimed to reduce this mechanistic knowledge gap. In this collection there are 10 papers providing new data on the epidemiology, neuropathologic features, immunopathogenesis, treatment and outcome associated with common or emerging inflammatory diseases affecting the nervous system of dogs and cats around the world.

Gonçalves et al. provide evidence that known or suspected immune-mediated conditions such as meningoencephalitis of unknown origin (MUO) and steroid-responsive meningitis-arteritis (SRMA) account for a substantially larger proportion of inflammatory nervous system diseases affecting dogs in the United Kingdom compared to infectious etiologies (3). In this study evaluating 1,140 dogs, they identified several risk factors associated with the signalment, history, and examination findings that aid in prioritizing infectious over immune-mediated differential diagnoses of inflammatory neurologic disease using multivariable and multinomial logistic regression.

The case series by Zdora et al. and Nessler et al. introduce variants of canine MUO with novel histological features, expanding the described repertoire of presumptively immune-mediated meningoencephalitides of dogs with distinct neuropathological phenotypes (3). Their collective findings highlight the heterogeneity of the conditions classified under the MUO umbrella that make conducting controlled and evidenced-based studies of this condition very difficult, reinforce the concept that MUO likely represents a spectrum of diseases in which the immune system reacts to different targets within the brain, and challenge conventional wisdom by suggesting the widely recognized necrotizing and granulomatous subtypes of MUO are perhaps representative of a disease continuum rather than distinct etiological entities.

In the continuing quest to elucidate autoimmune triggers of MUO, Barber et al. demonstrate that viral genomic material could not be recovered from the cerebrospinal fluid (CSF) from 98% (168/172) of North American dogs with neurological dysfunction and inflammatory CSF. These data add to existing literature supporting that occult viral infections are either not a common cause of MUO in dogs or that the genomic screening techniques used to date are sufficiently insensitive to detect these pathogens (4). Another study contributed by Barber and Koos did not demonstrate a functional or survival benefit when cytosine arabinoside treatment was added at the time of diagnosis of MUO to dogs chronically treated with cyclosporine and prednisone.

Two studies in this collection offer initial insights into the immunopathogenesis of SRMA and MUO. The proof-of-concept study by Wohlsein et al. demonstrates the existence of neutrophil extracellular traps (ETs) in the meninges and perivascular tissues of dogs with SRMA. Given the evolving importance of ET's in other canine immune-mediated diseases, the discovery of ETs in SRMA provides justification to further explore their role in the etiopathogenesis of this common disorder (5). Barber and Barber provide data derived from dog blood further implicating the T helper type 17 signaling pathway in the pathogenesis of MUO (6). These promising results encourage the continued exploration of peripheral blood-based biomarkers to assist with the diagnosis and treatment of this syndrome (1).

Kleeb et al. provide a timely update on the clinical manifestations and outcomes in dogs associated with the emerging Eurasian zoonotic viral disease, tick-borne encephalitis (TBE). They show that TBE in dogs originating from an endemic region in Europe bears striking similarities to the disease in humans with respect to the temporal evolution of constitutional signs of illness, its ability to cause protean neurological presentations, and the chronic neurologic disability experienced by a significant proportion of dogs that survive the initial illness. While TBE remains a life-threatening disease in dogs, prompt hospitalization and symptomatic treatment may prevent long-term complications and 67% of dogs survived in this study, which represents a considerable improvement in outcome compared to previous reports (7).

In companion articles, van Renen et al. and Kolb et al. thoroughly annotate the clinical, electrophysiologic, and neuropathologic features of a large cohort of cats with presumed immune-mediated polyneuropathy, an increasingly recognized condition in Europe that recapitulates many clinicopathologic features of human chronic inflammatory demyelinating neuropathy. These studies provide important data regarding clinical variables that are significantly associated with neurological recovery, histopathological findings in nerve biopsies that may be predictive of recovery, and the overall favorable outcome experienced by nearly 80% of cats with this condition.

Although the data provided by the papers in this collection will undoubtedly assist clinicians and pathologists in the diagnosis and management of these diseases, this topic reinforces the many facets of neuroinflammatory diseases that require additional investigation in order to further improve our understanding of, and develop better treatments for, these disorders.

## Author contributions

JR and AT contributed to drafting and proofing of this editorial. Both authors approved the submitted version.

## Conflict of interest

The authors declare that the research was conducted in the absence of any commercial or financial relationships that could be construed as a potential conflict of interest.

## Publisher's note

All claims expressed in this article are solely those of the authors and do not necessarily represent those of their affiliated organizations, or those of the publisher, the editors and the reviewers. Any product that may be evaluated in this article, or claim that may be made by its manufacturer, is not guaranteed or endorsed by the publisher.

## References

[1] Andersen-Ranberg E Berendt M Gredal H. Biomarkers of non-infectious inflammatory CNS diseases in dogs - where are we now? Part I: meningoencephalitis of unknown origin. Vet J. (2021) 273:105678. 10.1016/j.tvjl.2021.105678 34148601

[2] Gross S Fischer A Rosati M Matiasek L Corlazzoli D Cappello R. Nodo-paranodopathy, internodopathy and cleftopathy: target-based reclassification of Guillain-Barre-like immune-mediated polyradiculoneuropathies in dogs and cats. Neuromuscul Disord. (2016) 26:825–36. 10.1016/j.nmd.2016.08.015 27743643

[3] Cornelis I van Ham L Gielen I De Decker S Bhatti SFM. Clinical presentation, diagnostic findings, prognostic factors, treatment and outcome in dogs with meningoencephalomyelitis of unknown origin: a review. Vet J. (2019) 244:37–44. 10.1016/j.tvjl.2018.12.007 30825893

[4] Nessler JN Jo WK Osterhaus AD Ludlow M Tipold A. Canine meningoencephalitis of unknown origin- the search for infectious agents in the cerebrospinal fluid via deep sequencing. Front Vet Sci. (2021) 8:645517. 10.3389/fvets.2021.645517 34950723 PMC8688736

[5] Jeffery U Kimura K Gray R Lueth P Bellaire B LeVine D. Dogs cast nets too: canine neutrophil extracellular traps in health and immune-mediated hemolytic anemia. Vet Immunol Immunopathol. (2015) 168:262–268. 10.1016/j.vetimm.2015.10.014 26574161

[6] Park ES Uchida K Nakayama H. 2013 Th1-, Th2-, and Th17-related cytokine and chemokine receptor mRNA and protein expression in the brain tissues, T cells, and macrophages of dogs with necrotizing and granulomatous meningoencephalitis. Vet Pathol. (2013) 50:1127–34. 10.1177/0300985813488957 23651736

[7] Tipold A Fatzer R Holzmann H. Zentraleuropäische zeckenenzephalitis beim hund. Kleintierpraxis. (1993) 38:619–28.

